# Are the mutans streptococci still considered relevant to understanding the microbial etiology of dental caries?

**DOI:** 10.1186/s12903-018-0595-2

**Published:** 2018-07-31

**Authors:** Jeffrey A. Banas, David R. Drake

**Affiliations:** Iowa Institute for Oral Health Research, University of Iowa College of Dentistry, N406A DSB, Iowa City, Iowa, 52242 USA

**Keywords:** Dental caries, Mutans streptococci, Etiology

## Abstract

The mutans streptococci were once the primary focus of research dedicated to understanding the etiology of dental caries. That focus has now shifted to an emphasis on the ecological balances and complexities within the entirety of the plaque microbiome. Within that framework there are considerable differences of opinion regarding the importance and relative contributions of the mutans streptococci. This article explores the basis for the various viewpoints, the limitations of current knowledge, and the confounders that make it difficult to arrive at a consensus.

## Background

Are the Mutans Streptococci MS; (Table 1) still considered relevant? The answer will likely depend upon whom is asked. If personal experience is a guide, the answers will range from yes, the MS remain the most prominent cariogenic species identified, to no, in the absence of MS other acidogenic species fulfill the same role. Debating the subject can be an interesting academic exercise but differences in opinion can also have serious implications related to research priorities, clinical strategies and educating the next generation of dental health professionals. This article discusses some of the challenges to acquiring a detailed understanding of the microbial contributions to caries, the basis for divergent opinions on the relative importance of the MS, and the potential that remains for improving caries prevention and treatment based on a prominent role for the MS.

**Table 1:** Mutans Streptococci

Species	Host (s)
*Streptococcus mutans*	Humans
*Streptococcus sobrinus*	Humans
*Streptococcus criceti*	Rats and (rarely) Humans
*Streptococcus downei*	Monkeys
*Streptococcus ferus*	Rats
*Streptococcus macaccae*	Monkeys
*Streptococcus ratti*	Rats and (rarely) Humans
*Streptococcus hyovaginalis*	Swine

The search for an understanding of the causes of tooth decay spans millennia. The ancient Greek philosopher and scientist Aristotle is credited with linking the consumption of figs and sweet foods with the development of decay. With the recognition of a microbial component as observed by Leeuwenhoek in the seventeenth century and put forth in the chemico-parasitic theory by W.D. Miller in the 1880s, many efforts focused on the possibility of linking dental caries with specific microbial pathogens. J. Kilian Clarke [1] first proposed a role for *Streptococcus mutans* in 1924. Not long afterward, Stephan and Miller [2] provided a compelling demonstration of plaque composition differences between sound and carious enamel sites by measuring changes in pH over time following a glucose rinse. Presented in what are now commonly referred to as ‘Stephan curves’ the patterns of pH decreases and recovery by plaque organisms fundamentally differ depending on the clinical status of the sites associated with the plaque. Plaque from carious lesions is more acidogenic than plaque from sound enamel sites. Thus, the concept of a caries-related plaque dysbiosis or imbalance of acidogens, later to form the central core of the Ecological-based Hypotheses [3–5], was suspected prior to widespread interest in the MS. The key uncertainty was whether there was a consistent basis or microbial specificity behind caries-related dysbioses. That uncertainty appeared to be resolved once the MS started to receive increased attention.

A wealth of studies employing a variety of subject groups and study designs supported correlations between levels of the MS and all types of caries [6, 7]. In addition, the MS had properties that could explain the epidemiological connection between sucrose consumption and caries, as well as its leading role in initiating the caries process in a manner consistent with the acid theory of enamel demineralization. Embodied within the Specific Plaque Hypothesis [6], the idea was that if dental caries were to be treated as a specific infection, for example by specifically targeting the MS, then a vaccine could be developed and preventive and treatment measures would make tremendous advances. Dental caries would not be eliminated but its occurrence would be significantly reduced.

However, not everyone was convinced. Some felt that greater emphasis on the contributions from the breadth of the microbiome would lead to more effective means of caries prevention [3]. Kleinberg [4] modeled Stephan’s curves to argue the importance of microbial base production while noting instances of caries that could not be attributable to the MS and examples where relatively high proportions of MS were recovered from sound enamel. Kleinberg also questioned whether levels of MS associated with caries were sufficient to be the major determinants responsible for the pathology. Takahashi and Nyvad [5, 8] promoted the idea that non-MS acidogens preceded and promoted the ascendancy of MS. These various formulations of Ecological Plaque Hypotheses were improvements that more explicitly recognized the combined effects of the plaque microbiota and the changes to the microbial profile that occurred during different stages of caries development. Within the framework of the Ecological Plaque Hypotheses, however, there remain differences of opinion regarding the relative contributions of the MS. There are several reasons for these differences of opinion, among them that the determination of the ‘cariogenic microbiome’ is not nearly as straightforward as might be presumed (Table 2) and inconsistencies in the data have left plenty of room for speculation.

**Table 2:** Summary of obstacles to determining the microbial etiology of caries

Obstacle or Confounder	
Sampling Choices	
- saliva instead of dental plaque - plaque pooled from sites of caries and intact enamel - microbiology of carious sites can vary considerably over small distances	
Substrate Susceptibility	
- tooth sites may vary in susceptibility to dental plaque of similar composition	
Caries Process	
- the microbiology of caries evolves as the lesion progresses in severity	
Genetic Contributions	
- salivary composition and constituents may elevate or depress caries risk	
Determination of the Plaque Microbiome	
- culture-based methods have a narrow focus - next generation sequencing outcomes may differ from lab to lab analyzing the same sample - 16S gene-based sequencing poorly differentiates species of oral streptococci	
Biological Significance	
- little evidence for what constitutes biologically significant levels of individual plaque species	
Caries Incidence	
- the microbial basis for occasional caries may typically differ from that for rampant caries	

## Main text

### The multifactorial nature of caries etiology

Experiments with germ-free rats demonstrated that caries will not form in the absence of a plaque microflora [9]. But the presence of a plaque biofilm and its composition are far from the only variables that affect caries risk. As numerous investigations have revealed over the years, salivary flow, salivary composition, diet, nutrition, parental education level, transmission and age of acquisition of cariogenic species, oral hygiene practices, fluoride exposure, tooth anatomy and enamel composition are just some of the variables that intertwine with the plaque microflora to shape caries risk. Further complicating matters, the crystal structure of the substrate – enamel – may lack homogeneity, varying among individuals and perhaps even from site to site within a tooth, affecting local sensitivity to acid [10]. A similar scenario may exist for fluoride incorporated into enamel [11]. The influence of host genetics has also received considerable attention. Some polymorphisms, such as those found for the salivary factor lactoferrin [12, 13], may influence caries risk via an effect on the composition of the plaque microbiome or members therein. But many other genetic associations with caries involve enamel development [14] and are more likely to affect tooth sensitivity to acid demineralization. Vieira et al. [15] used an animal model to show that the amount of amelogenin during enamel development correlated with sensitivity to acid dissolution. If enamel sites differ in sensitivity to acid, as it appears they may, then it can be reasoned that the microbial challenge necessary to initiate and perpetuate caries need not be equivalent at all sites. Taken further, an equivalent microbial challenge may be sufficient to initiate caries at one site but not at another. As a general principle it seems reasonable to suggest that the more acid-sensitive an enamel site is, the more diverse the microbial challenges can be that are capable of bringing about a similar result, in this case a carious lesion.

As noted above, one argument for removing the spotlight from the MS is that they sometimes cannot be detected at sites of decay and conversely, are sometimes well-represented on sound enamel. But with the above considerations in mind, these anomalies may be explained by instances where substrate (localized tooth enamel) sensitivity plays an essential role in shaping caries risk. When teeth, or sites within teeth such as non-fully formed fissures, are abnormally sensitive to acid challenge the situation is analogous to an immunocompromised host who is at risk of disease from opportunistic pathogens within the normally mutualistic microflora. The key to understanding disease acquisition in these instances is found more in recognizing the contribution of host susceptibility than focusing solely on the microbial challenge.

Many investigations of the microbial basis of caries examine individuals who exhibit the most severe forms of the disease, in particular children who develop caries very early in life. Intuitively this makes sense as these individuals would benefit most from progress in treatment and prevention. But one can also speculate whether genetic predispositions [16] to caries are overrepresented among these individuals. Nutritional concerns, linked to the known risk factor of low socio-economic status, and enamel hypoplasia may also be prevalent among these children. If true, links to strong cariogenic species may not be necessary to bring about severe caries, but when strong cariogens are present they may make the development of early/severe caries nearly inevitable.

The point to be made is that it is clearly challenging to control for so many variables in order to zero in on the relative contributions of the plaque microbiome or unique members of that microbiome. Consequently, it may be unrealistic to expect to find perfect associations between caries and microbial agents even if a single species were to be responsible for the vast majority of caries involving properly formed teeth. If genetic influences are eventually found to be widespread, then it may be reasonable to question the amount of effort dedicated to focusing attention on the microbial challenge. But that point has not been reached. Identifying imperfect associations of the plaque microflora with caries may still represent excellent opportunities to significantly improve treatment and prevention options.

## Sampling the cariogenic microbiome

Conceptually, identification of the cariogenic microbiome seems to be a simple matter of comparing the microbial compositions of plaque at sites of caries and sound enamel. But most studies don’t do that. A surprising number of studies rely on saliva samples which have a markedly different microbiome than plaque [17], and many other studies pool plaque samples to one degree or another. Thorough plaque collection at a particular site may be beneficial as the microbiome may vary significantly over very minor distances [18, 19]. For example, Duchin and van Houte [18] sampled incipient (white-spot) lesions and recorded up to a 100-fold difference in the recovery of *S. mutans* from samples taken from a single lesion. Pooling on a larger scale, often done for logistical and economical reasons, may nonetheless risk diluting out the differences one is attempting to identify.

Pooling samples is particularly common among contemporary culture-independent studies [20–27] that do so in part to obtain sufficient microbial DNA. Some studies pool plaque from both healthy and carious sites [22, 25, 27], preferring to look for evidence of global differences between the microbiomes of subjects with caries or those who are caries-free. This approach may be most effective if the microbiomes of sound enamel sites differ between caries-free subjects and subjects with caries. But it is uncertain that they do. Even pooling plaque from exclusively sound or carious sites is not without potential complication. The microbiomes associated with caries may vary by the type or anatomical location of the lesion and evolve as the lesions progress in severity [19, 28]. Additionally, the microbiome varies considerably from one individual to the next [29] which can make it difficult to pinpoint relevant caries-related differences in studies that pool data from multiple subjects.

Given the issues associated with plaque collection, it can again be expected that attempts to find associations between particular bacterial species and caries will be imperfect no matter how strong the role proposed for the putative cariogenic species. Yet, in spite of these obstacles, most culture-independent studies in fact confirm a link between the MS and caries when analyses are taken to the species level [20, 22, 24, 25, 27]. Other associations exist, especially for species of *Bifidobacterium*, *Scardovia*, *Veillonella* and non-MS species of *Streptococcus* [20, 22, 23, 26, 30], but among these the streptococci, perhaps in conjunction with *Veillonella*, are the organisms most strongly linked to the earlier stages of caries development.

## Culture-based versus culture-independent studies

The case for MS involvement in dental caries was built largely upon culture-based studies during a time that preceded the technical advancements that made possible determinations of the complete microbiome. Culture-based studies were necessarily limited in scope and typically tracked the MS and possibly lactobacilli, select non-MS streptococcal species, or species of *Actinomyces*. When it became apparent that the majority of unique taxa in the oral cavity cannot be cultured in vitro, it reinforced the skepticism some shared towards ceding such a prominent role for the MS. What is frequently underemphasized, however, is that over 80% of the plaque *biomass can* be cultured in vitro [31] and that culture-independent studies have not typically revealed novel associations between caries and non-culturable taxa that match those of the MS.

Also rarely discussed is that culture-independent studies, like their culture-based counterparts, are subject to significant potential experimental error. In culture-based studies, sample storage can introduce experimental error as can imperfect selective and differential media for the isolation of MS [32] or other species of interest. Most culture-independent surveys of the plaque microflora have used a 16S rRNA gene-based approach for cataloguing microbial diversity. This approach is limited to defining operational taxonomic units (OTUs); some species associations can be made with individual OTUs but in general species assignment is limited [33–35]. This shortcoming is even more pronounced for the oral streptococci because they are poorly differentiated by 16S rRNA gene sequences [36]. Another area of uncertainty is the accuracy of the results. Hiergeist et al. [37] tested the consistency of 16S rRNA gene-based determinations of the gut microbiota by sending portions of the same stool sample to nine different laboratories. Although each laboratory displayed reproducible results in technical replicates, the proportions of each taxa reported from laboratory to laboratory varied widely. DNA isolation methods accounted for some variability, but even when that was controlled there were some stunning differences. For example, the proportions of Actinobacteria varied between less than 1% to nearly 25%, the proportions of Bacteroidetes from 6 to 38%, and the proportions of Firmicutes from 45 to 82%. It is reasonable to believe that similar levels of variability may be inherent in analyses of the oral microbiome.

These considerations may explain some of the variability among culture-independent studies in associating particular taxa with caries or health, and may also explain apparent contradictions. Levels of *Streptococcus sanguinis* have been found to correlate with health in most studies [20, 22, 24] but with caries as well [25]. As before, these considerations reinforce the theme that an expectation of perfect species associations is unrealistic. The fact that consistent associations with the MS are found anyway seemingly provides a stronger argument for a prominent role in caries than do the exceptions cited when arguing against a major role for the MS.

## Critical thresholds of representation

The various culture-based and culture-independent studies that have correlated levels of species or taxa with caries typically report those levels as means or medians. But these data may be accompanied by large standard deviations due to substantial variability among samples or subjects. Many of those who have looked critically at MS associations with caries have raised the important issue of the threshold of representation necessary for a single species or group of species to make a biological impact. It is the area of cariology for which there may be the greatest deficit of experimental information.

It is logical to presume that the threshold representation necessary to contribute to caries development is proportional to the strength of the cariogenicity properties of any given species. Acid production is the most obvious property but it is not the only one. What has boosted the MS resume is that they excel at the *rate* of acid production [38], the low pH at which they continue to produce acid [38], and the synthesis of extracellular glucans that promote acid diffusion [39]. A similar narrative has not been constructed around alternative acidogens with the possible exception of non-MS low pH streptococci [8]. Using a mixed-species plaque model in rats, van der Hoeven and Franken [40] found that the addition of *S. mutans* – which attained a plaque representation of 20% -- yielded significantly higher amounts of lactic acid than the mixed-species plaque without *S. mutans*. Conversely, Kleinberg [4] concludes that the presence of an acidogen at 1 to 2% or less will not significantly impact the local pH. Since there are instances of MS levels at carious sites within this range or lower, it is cited as evidence that the MS cannot independently bring about caries.

As important as these observations are, it is arguably more relevant to consider ‘typical’ MS representation as the scientific and methodological explanations for exceptions have already been discussed. When considering the ‘typical’ representation of MS, it should be noted that the same limitations cited in the section on sampling are applicable here as well. Consequently, there are a limited number of studies that quantify MS from strictly carious sites and report the data as a percent of the total microflora. The available data indeed display quite a range of values and underscore the basis for why differences of opinion exist regarding the role of the MS. It should also be noted that the percentages reported will be proportionally lower if one accounts for the non-cultivable portion of the microflora. Loesche et al. [41] found MS levels ranging from undetectable to over 30% of the total cultivable microflora in both caries-free children and children with rampant caries but the children with caries were statistically more likely to have higher levels. Huis in’t Veld et al. [42] reported that tooth surfaces with greater than 5% *S. mutans* were the most likely to become carious over a period of 10 months. Meiers et al. [43] reported that *S. mutans* from carious fissures averaged 7.3% of the total cultivable count. More recently, Hughes et al. [44] reported *S. mutans* as high as 10% of the total blood agar count from molars of children with severe early childhood caries.

A couple studies stand out for data that fit a broader pattern. Loesche and Straffon [45] found that the average *S. mutans* representation in fissures of high caries-active subjects of 5 to 12 years of age was nearly 25% whereas the average proportion in low caries-active subjects was just 0.1%. The culture-independent microbiome study by Johannson et al. [27] found a correlation between MS and caries for a high caries prevalence Romanian cohort but not with a low caries prevalence Swedish cohort. Caries have historically been associated with dietary carbohydrates, but the introduction of processed sugars really accelerated rates of decay [46, 47]. Despite ongoing concerns with high levels of dietary sugars, the caries experience in wealthier countries declined greatly in the years following the widespread incorporation of fluoride into water supplies and dental hygiene products [48]. This may reflect an ability of fluoride, perhaps in conjunction with other preventive measures, to significantly moderate the cariogenic process [46] and as a result act as a confounder of efforts to link caries with a consistent microbial source. This may also help explain why low levels or the absence of detectable MS are better predictors of future health than high levels of MS are predictors of future caries [49–51]. It is also possible that patterns of consumption of sucrose in its most cariogenic forms [52] have changed. Much of the research linking the MS to caries occurred during the zenith of caries prevalence and incidence in Western nations. It may be that those are the conditions under which the MS exert the greatest influence and still do among individuals who habitually consume cariogenic foods and beverages and lack effective oral hygiene practices.

## Is an all-encompassing hypothesis of caries etiology necessary?

The current consensus in the oral biology community favors some form of ecological-based hypothesis [3, 4, 8]. This is entirely appropriate in spite of what we would consider misconceptions regarding how the MS were integrated within The Specific Plaque Hypothesis. Still, most adherents of the Ecological Plaque Hypotheses acknowledge some level of specificity or reproducibility to the taxa associated with decayed surfaces. Rosier et al. [53] chronicle the development of hypotheses for the etiology of oral diseases and conclude that none of the current versions adequately explain all facets of disease development and progression. Perhaps that is because there are multiple pathways to caries that make it difficult to place all possibilities and circumstances under a single umbrella, and insisting on doing so may in fact detract from efforts to substantively improve prevention measures for a sizeable subset of the at-risk population.

There are intriguing aspects of caries etiology that have not been adequately explored. One of those is potential synergy between *S. mutans* and *S. sobrinus* as caries experience tends to be worse when both are isolated from carious lesions [54–56]. Another is the contribution of non-MS low pH streptococci which may be among primary plaque colonizers, numerically dominant to the MS (potentially addressing the concern about proportional representation of the MS) and able to set the stage for the ascension of stronger cariogens [8, 57]. The idea of the MS as ‘keystone’ pathogens [58] is also worthy of investigation. In this context, the appropriate analogy may be sports related. No Hall of Famer could win a game without teammates. But the supporting cast around a Hall of Famer can vary while still enjoying a consistent level of success. But take away the Hall of Famer and the supporting cast is a much diminished entity. It doesn’t mean they can’t win, but their chances of doing so are significantly less. In this analogy, the MS are the star players. They make caries much more likely though not a certainty. Supporting players, the other plaque members, make significant contributions but the specificity of those plaque members is not nearly as critical. Finally, caries still occur in the absence of the star play, the MS, but are more likely to be part of a pattern of low caries occurrence.

It has been suggested that the particular species composition of dental plaque is less important than its collective protein expression profile [59]. This would be one way to explain how caries develop from diverse plaque microbial profiles. However, this model doesn’t necessarily preclude a more prominent role for certain species as described in the sports analogy above.

The importance of understanding how caries develop is intimately linked to strategies for treatment, prevention, diagnosis, and risk assessment. We believe that caries have multiple etiologies, but that the MS often make a critical contribution. Current preventive strategies, however, remain largely non-specific. In the heyday of the Specific Plaque Hypothesis, considerable effort was directed towards reducing or eliminating *S. mutans*. The principle of using antibiotics to treat decay as a specific infection met with apparent success [60], irrespective of the practicality of widespread administration of antibiotics for caries prevention. However, the success rate, an approximately 46% reduction in new lesions, appeared to be matched at the time by use of chlorhexidine [61], a non-specific anti-plaque agent, which in more recent times has been found to be of questionable efficacy in caries prevention [62, 63]. Better evidence for the efficacy of targeting the MS may be found from studies of the sugar substitute xylitol. Xylitol, a sugar alcohol to which *S. mutans* is particularly sensitive, has been found to help prevent caries in a manner that coincides with reductions in the levels of MS [63]. There are also instances where the prevention or delaying of the acquisition of *S. mutans* in young children has met with rather striking success. Kӧhler and Andréen [64] reported follow-up results 19 years after mothers were treated to delay acquisition of *S. mutans* by their children: 7 of 19 interventional children were *caries-free* at age 19 compared to 0 of 28 control subjects. Success appeared to be dependent on delaying acquisition of *S. mutans* beyond 3 years of age whereas no benefit was evident if *S. mutans* could be detected prior to that threshold. These observations may help explain instances where treatment of mothers to prevent caries in their children didn’t meet with similar success [65] and simultaneously reveals room for improvement in strategies to prevent MS transmission/acquisition and the potential benefits from successfully doing so.

The pinnacle of success, based on targeting the MS, was to be development of a caries vaccine. Proof of principle was demonstrated by a large body of work in animal models. Quite a few human trials were also carried out, mostly using nasal, oral or passive immunization strategies, but none of those trials measured caries rates as the outcome (Table 3). Since prospective vaccines were based on immunizing against the MS, there has been a loss of momentum in reaching this goal that has paralleled the diminished perception of the importance of the MS. Risk aversion associated with active vaccination for a non-life-threatening disease may have also played a role but less risky passive immunization strategies harbor considerable promise. The decline in caries vaccine efforts is especially unfortunate as, collectively, the field was at the threshold of taking a major step towards answering whether, and to what extent, targeting the MS could reduce caries rates. Opinion and speculation, however adamant, are no substitutes for experimental verification.

**Table 3:** Dental caries vaccine trials using human subjects

Study	Immunization Route	Outcome Measure
Krasse et al.; 1978	Topical	*S. mutans* specific IgA
Mestecky et al.; 1978	Oral	*S. mutans* specific IgA
Gahnberg et al.; 1983	Oral	*S. mutans* specific IgA; Recovery of *S. mutans*
Cole et al.; 1984	Oral	Antibody response; Recovery of *S. mutans*
Gregory et al.; 1987	Oral	*S. mutans* specific IgA; Recovery of *S. mutans*
Czerkinsky et al.; 1987	Oral	*S. mutans* specific IgA or IgM
Smith et al.; 1987	Oral	*S. mutans* specific IgA; *S. mutans* to total strep ratios
Ma et al.; 1987	Passive	Recovery of *S. mutans*
Smith and Taubman; 1990	Topical	Antibody against GTF; *S. mutans* to total strep ratios Recolonization with *S. mutans*
Ma et al.; 1990	Passive	*S. mutans* colonization
Childers et al.; 1990	Oral	Specific salivary IgA; Plasma IgG response
Filler et al.; 1991	Passive	Recovery of *S. mutans*
Childers et al.; 1997	Nasal	Specific salivary, nasal and serum IgA, IgM, and IgG
Hatta et al.; 1997	Passive	*S. mutans* to total strep ratios
Ma et al.; 1998	Passive	Protection against colonization
Childers et al.; 1999	Nasal	Specific salivary, nasal and serum IgA and IgG
Shimazaki et al.; 2001	Passive	Recolonization of *S. mutans*
Childers et al.; 2002	Nasal	Specific salivary, nasal and serum IgA and IgG

Caries prevention based on the Ecological Plaque Hypotheses is predicated on reversing the imbalance of acidogens. However, the mostly non-specific oral hygiene measures routinely in use often fail to bring about sustainable corrections of ecological imbalances. Newer strategies for prevention, or revived strategies such as treatment with silver compounds, may have implications for plaque ecology. Silver’s caries arresting properties are likely due to its strong antibacterial activity. Whether it promotes long-term changes in plaque ecology is unknown [63]. Probiotics, in their infancy for oral applications, may alter plaque ecology but they are frequently chosen based on antagonism toward *S. mutans* and/or evaluated for effects on oral levels of MS [66]. However, there are examples of modest efficacy even in the absence of an understanding of how the benefit is manifested [67, 68]. The use of arginine as a prebiotic to stimulate the production of acid-neutralizing alkali may eventually select for a health-related microbiota [69]. Similar to trials with select probiotic regimens, the incorporation of arginine into toothpastes has been found to result in modest but statistically significant reductions in caries rates [70]. However, in at least one study [71], a beneficial effect required two years to become evident (no difference was noted at the one-year mark) possibly indicating that the pace of altering the microbiota is relatively slow. It is precisely this type of scenario in which complementary targeting of strong cariogenic species might yield the greatest benefit.

## Conclusions

The predominant model of caries etiology has transitioned from the Specific Plaque Hypothesis to Ecological Plaque Hypotheses but controversy continues regarding the role played by the MS. We have argued here that the data remain consistent with a prominent role for the MS in caries development, especially among those who experience the disease at its worst. Accordingly, we believe it is essential to continue to consider the role of the MS in the multifactorial caries process in order to bring about effective preventive and clinical treatments.

## Abbreviations

MS: mutans streptococci
OTU: operational taxonomic unit
rRNA: ribosomal RNA
